# Use of Visual Electrophysiology to Monitor Retinal and Optic Nerve Toxicity

**DOI:** 10.3390/biom12101390

**Published:** 2022-09-29

**Authors:** Tsun-Kang Chiang, Kayla Marie White, Shree K. Kurup, Minzhong Yu

**Affiliations:** University Hospitals Eye Institute, Case Western Reserve University, Cleveland, OH 44101, USA

**Keywords:** toxicity, drug, electrophysiology, electroretinogram, electrooculogram, visual evoked potential, eye, retina, optic nerve, functional exam

## Abstract

It is important for clinicians to consider exposure to toxic substances and nutritional deficiencies when diagnosing and managing cases of vision loss. In these cases, physiologic damage can alter the function of key components of the visual pathway before morphologic changes can be detected by traditional imaging methods. Electrophysiologic tests can aid in the early detection of such functional changes to visual pathway components, including the retina or optic nerve. This review provides an overview of various electrophysiologic techniques, including multifocal electroretinogram (mfERG), full-field ERG (ffERG), electrooculogram (EOG), pattern electroretinogram (PERG), and visual evoked potential (VEP) in monitoring the retinal and optic nerve toxicities of alcohol, amiodarone, cefuroxime, cisplatin, deferoxamine, digoxin, ethambutol, hydroxychloroquine, isotretinoin, ocular siderosis, pentosane, PDE5 inhibitors, phenothiazines (chlorpromazine and thioridazine), quinine, tamoxifen, topiramate, vigabatrin, and vitamin A deficiency.

## 1. Introduction

It is not uncommon for the earliest manifestation of drug toxicity to be detected in the ophthalmology clinic. In this situation, it often is a complex picture of clinical tools, drug exposure, and electrophysiologic abnormalities that point to the offending agent. Often, patients can be on multiple potentially retina-toxic or neurotoxic medications, and establishing probabilities can be helped by advances in electrophysiologic testing. Here we discuss drugs that are widely used currently (or were in the past) that are common offenders. The testing techniques must be used in combination with a very skillful ocular exam designed to quantitatively detect subtle abnormalities by appropriately qualified and trained observers.

While this list is not exhaustive, the testing methods allow a discussion of the current options available to ensure that a proper diagnosis is made. We examine the ocular implications of a variety of widely used medications, which can be seen listed with generic and brand names in Table 1. Occasionally, a drug may be lifesaving, and careful assessment of the risk versus benefits must be made. The current drugs discussed will also allow broad application potentially of similar techniques to detect similar toxicity of newer analogues. Screening for toxicity may not be the best terminology used in these applications, and detection of toxicity is a rather more appropriate term. We now discuss the agents commonly implicated for ocular toxicity in the clinic.

## 2. Alcohol

Alcohol intoxication from the ingestion of methyl alcohol can cause various forms of vision dysfunction, ranging from acute optic neuropathy to fetal alcohol syndrome. Alcohol may also contribute to or alter the development of other ocular pathologies such as age-related macular degeneration, diabetic retinopathy, cataract, and glaucoma [1]. Here we review changes in electrophysiology related to the processes attributed mostly to alcohol ingestion.

Methyl alcohol ingestion can cause damage to the optic nerve and retina through the accumulation of toxic metabolites of formaldehyde and formic acid [1]. Patients may present with significantly reduced visual acuity and optic disc hyperemia, edema, or pallor [2,3]. In acute intoxication episodes and chronic wine alcoholism, scotopic ffERG findings include diminished a-wave and b-wave amplitudes [3,4]. Similarly, in mice exposed to methyl alcohol, both scotopic and photopic ERGs have reduced a-wave and b-wave amplitudes [5]. VEP was found to be abnormal in about 40% of patients with methanol exposure [6]. The most common abnormalities on VEP were increased implicit times followed by decreased N1P1 and P1N2 amplitudes [6].

Ninety percent of patients born with fetal alcohol syndrome (FAS) will have ocular manifestations of the disease [7]. Common facial dysmorphic features include short palpebral fissures, telecanthus, epicanthus, and blepharoptosis. Key functional structures of the eye may be impacted, with common findings of optic nerve hypoplasia and strabismus in these patients [8]. On scotopic ffERG, b-wave amplitudes are severely reduced in more than 90% of the patients with FAS. Increased a-wave and b-wave implicit times and reduced a-wave amplitude are also seen in a mixed rod–cone response to a bright white flash. Compared to gender- and age-matched controls, FAS patients also have a reduced Rmax (maximal b-wave amplitude) and increased log(k) (retinal sensitivity) on the intensity–response series [8]. Photopic ERG is less affected compared to scotopic ERG [8].

## 3. Amiodarone

Amiodarone is a class III antiarrhythmic medication that is used in the treatment of recurrent coronary rhythm abnormalities including ventricular tachycardia or fibrillation. It prolongs the actional potential of cardiac myocytes by blocking the potassium channel in cardiac tissues. It is associated with significant systemic adverse effects on the thyroid, pulmonary system, and eye that are most likely due to the accumulation of the medication in other tissues such as the retina [9,10]. The most common ocular dysfunction associated with amiodarone is corneal verticillata, a vortex-shaped pattern of corneal epithelial deposits which causes opacification and can lead to vision loss [11]. Retinopathy is only infrequently seen in patients taking amiodarone, and there is no definite evidence associating the medication with retinal toxicity [11,12]. Similarly, no significant changes were found on ffERG and mfERG. While subnormal results were reported on mfERG, no clear pattern of changes appeared in patients taking amiodarone [12].

## 4. Cefuroxime

Cefuroxime is a broad-spectrum cephalosporin antibiotic that is commonly used in ophthalmic surgery for prophylaxis to reduce the risk of post-operative infection. Intracameral cefuroxime and subconjunctival cefuroxime are commonly used during cataract surgery and trabeculectomy surgery, respectively [13]. It achieves its bactericidal effect through the inhibition of bacterial cell wall synthesis. Ocular adverse effects of cefuroxime include intraocular inflammation [14], macular infarction with cystoid macular edema [15], and cornea edema [16]. On optical coherence tomography (OCT), serous macular detachment and disruption of the ellipsoid zone have been reported in a few cases [13]. While still not fully understood, given that macular edema predominates in the outer nuclear layer, transient sodium–potassium pump dysfunction in the retinal pigment epithelium (RPE) might play a role in cefuroxime toxicity [14].

Electrophysiologic changes are also seen in cefuroxime toxicity, which can result from the RPE dysfunction from cefuroxime toxicity. On ffERG, reductions in a-wave and b-wave amplitudes in dark-adapted and light-adapted conditions have been reported [13,14,17]. On the other hand, a case series noted that photopic b-wave amplitudes were normal and only a reduction in scotopic b-wave amplitudes was noted [14]. The different results in photopic b-wave amplitudes may be related to the higher dose of cefuroxime in the case series (40–50 mg) compared to other studies with lower concentration (1–12.5 mg). Cefuroxime is also associated with reduced signals in all rings of mfERG [13]. Further investigations are needed to elucidate the relationship between cefuroxime dose and electrophysiologic changes.

## 5. Cisplatin

Cisplatin (cis-diamminedichloroplatinum) is a platinum-based chemotherapy agent that functions by alkylating DNA, widely used for the treatment of bladder, ovarian, and testicular cancer. It can be administered either intravenously or intraarterially, and both are associated with ocular toxicity. Cisplatin-related ocular toxicity may manifest as vaso-occlusive pathologies (e.g., central retinal artery occlusion, cilioretinal artery occlusion), [18,19] optic neuritis [20], or optic nerve ischemia. It is theorized that cisplatin causes retinal toxicity by producing excessive oxidative stress, and symptoms commonly include retinal ischemia [21,22].

On ffERG, patients with germinal cell cancer who were treated with intravenous cisplatin were found to have significantly reduced b-wave amplitudes and prolonged a-wave implicit time in testing for isolated cone response with 30-Hz flickering white light [23]. On VEP, cisplatin retinopathy is associated with increased implicit times and reduced amplitude. The effect on VEP appears to be independent of the cumulative dose of cisplatin received [24,25]. On the other hand, for patients with reversible cisplatin-related optic neuritis, VEP response was initially completely absent then returned with delayed implicit times [20].

Cisplatin administered as part of intra-arterial chemotherapy has also been associated with electrophysiologic changes. Case reports have found extinguished ERGs and diminished EOG in patients receiving cisplatin and BCNU (carmustine) or cisplatin alone [26]. In addition, VEP prolongation can be seen in patients with intra-arterial cisplatin either before or after visual acuity loss was detected [24].

## 6. Deferoxamine

Deferoxamine is a chelator for the treatment of chronic iron overload due to a variety of pathologies that require long-term treatment with blood transfusions. It can be used in the treatment of a variety of hematologic conditions that result in chronic anemia [27]. It functions as a chelating agent by forming a covalent bond with ferric iron to form ferrioxamine, which is eliminated by the kidney in urine.

Deferoxamine has multiple well-characterized ocular side effects, including retinopathy, cataract, and optic neuropathy [28,29]. While not completely understood, apoptotic changes in RPE cells have been linked to deferoxamine [27]. Patients can present with night blindness, decreased visual acuity, and abnormal color vision [27,29,30]. On ffERG, patients who received deferoxamine were found to have reduced a-wave and b-wave amplitudes in scotopic conditions. In addition, prolonged b-wave implicit time can be seen in photopic conditions [31]. On mfERG, statistically significant reduced retinal response density (RRD) can be seen centrally, while prolonged P1 implicit time can be seen in the peripheral area [31]. On EOG, reduced Arden ratio can be observed in patients treated with deferoxamine. However, there appeared to be large variability in the reduction of the light-peak to dark-trough ratio [28,32]. On VEP, two earlier studies reported a subset of cases with increased VEP implicit time after deferoxamine therapy for some months, even though an ophthalmological exam did not find any abnormality [29,33]. Recent studies, however, have found no statistically significant changes in both amplitudes and P100 implicit times on VEP for patients who received deferoxamine compared to controls [31,34].

## 7. Digoxin

Digoxin is a commonly used medication in cardiology for the treatment of heart failure and supraventricular arrhythmia. It reversibly inhibits the sodium/potassium ATPase pump in myocardial muscle cells and thereby increases myocardial contractility [35]. Digoxin-related ocular toxicity is most commonly associated with color vision deficiency, which can be seen in about 20–30% of patients [36]. Other common symptoms include blurred vision with or without dyschromatopsia and worse vision in bright light and flashing or scintillating lights [37]. Visual symptoms can often be seen days or weeks after drug initiation [35].

As several different retinal cells, including photoreceptors, RPE, and Müller cells, express the isoforms of sodium/potassium ATPase that are sensitive to digoxin, digoxin ocular toxicity can lead to RPE or photoreceptor dysfunctions that lead to electrophysiologic changes [36]. On ffERG, digoxin use has been associated with findings such as decreased b-wave amplitudes of cone-mediated response and prolonged b-wave implicit time [37,38]. The prolongation in b-wave implicit time is reversed after the discontinuation of the medication, which makes it feasible to use ERG to monitor for digoxin visual toxicity [38]. These changes may be related to the reversible inhibition of sodium/potassium ATPase by digoxin that disrupts ion transportation, which can lead to a prolonged period of repolarization on rods/cones and prolonged duration of action potential on retinal ganglion cells. mfERG showed a diffuse reduction in amplitudes in the central 10 degrees of the macula [35]. The EOG light-peak to dark-trough ratio is reported to be high during clinical toxicity, but it is likely to be normal [39].

## 8. Ethambutol

Ethambutol is an antimycobacterial medication that achieves its therapeutic effect through the inhibition of arabinosyltransferase. It prevents mycobacterial cell wall synthesis and is commonly used as part of the treatment for *Mycobacterium tuberculosis* in combination with other agents. The most commonly reported ocular side effect is ethambutol-related optic neuropathy (EON) and happens in about 1.3–1.5% of patients who are treated with ethambutol [40,41]. Patients with EON present with decreased visual acuity and deficits in color vision that are reversible after cessation of therapy. EON can be subdivided into the axial (central) form, associated with cecocentral scotoma, and the periaxial form, associated with changes in the peripheral visual field [42]. While the exact mechanism of ocular toxicity of ethambutol remains unclear, metal chelation by ethambutol, including zinc and copper, may be related as it causes disruption to mitochondrial homeostasis in retinal ganglion and neuronal cells [42].

Electrophysiologic changes reported with EON include a reduction in the difference of the maximum b-wave amplitude relative to the maximum a-wave amplitude between light adaptation and dark adaptation [43]; an increase in b-wave implicit time in light-adapted ffERG [44]; and a decrease in amplitude in pattern ERG [45]. On mfERG, significantly delayed implicit time of P1 (the peak of the first positive wave) in rings 4–6 was seen in patients with EON [46]. Other case reports reported reduced amplitudes of N1 (from the baseline to the trough of the first negative wave) and P1 (from the baseline to the peak of the first positive wave) near the macula [47,48], but these changes were not statistically significant in a larger study [46]. On EOG, supranormal Arden ratio can be seen in early toxicity; however, reduced Arden ratio can also be seen [49]. On VEP, delayed implicit time and reduced peak amplitudes can be seen in EON patients [45]. Specifically, a “scotomatous response” can be seen months after the start of ethambutol therapy, whereby the normal P100 signal is replaced by a paramacular positive–negative–positive response [50]. Taken together, the electrophysiology changes associated with ethambutol suggest that EON likely affects not only the optic nerve, but also the retinal pigmentary epithelium and the peripheral neurosensory retina.

## 9. Hydroxychloroquine

Hydroxychloroquine (HCQ, Plaquenil^®^) is an antimalarial medication also commonly used in the treatment of a variety of autoimmune diseases, including lupus, rheumatoid arthritis, and Sjorgren’s disease. It achieves its therapeutic effect through an increase in lysosomal pH and suppression of the activation of the innate immune system. The retinal toxicity of hydroxychloroquine and chloroquine has been studied extensively. Risk for retinal toxicity is associated with a higher dosage (>5 mg/kg) and has a reported 10% risk at 10 years of use [51]. The disease is characterized clinically by RPE abnormalities on OCT in early stages and “bull’s-eye” maculopathy in the later stage of the disease process [52]. The mechanism of HCQ’s ocular side effect remains uncertain but may be related to the disruption of RPE and/or photoreceptor metabolism [52]. Given the need for the immediate cessation of the medication once retinal toxicity is detected in the early stage of the disease to prevent future loss, routine ophthalmologic screening plays an important role in preventing visual loss due to HCQ use. Visual fields and OCT are the most common modalities in the current guideline for the screening for HCQ retinopathy [51]. However, as more emphasis is put on “objective” screening methods rather than “subjective” screening methods (i.e., visual fields), electrophysiology testing, especially mfERG, can play an important role in screening for the disease [52,53,54]. Furthermore, after discontinuation of HCQ, recovery of visual function can also be detected in the reversal of mfERG changes [55]. Systematic review also found mfERG to be a modality with 90% sensitivity and 52% specificity, which further supports the use of mfERG as an adjunct tool in the screening of HCQ retinal toxicity [53].

On mfERG, abnormal results associated with HCQ retinopathy include amplitude reduction, prolonged implicit time, reduction in ring response, and ring ratios greater than the normal limit. Additionally, color difference plots indicate decreased response density [53,56]. The amplitude reduction appears to be most commonly seen pericentrally as compared to full-field or central loss [54,57]. Therefore, compared to mfERG, ffERG appears to be less sensitive to HCQ retinopathy, and only a subset of cases with mfERG deficits have a reduction in the amplitude of ffERG [57]. These findings may be related to the different effects of HCQ on photoreceptors in different locations, but further work is necessary to elucidate the mechanism of HCQ ocular toxicity. On VEP of patients with HCQ toxicity, P100 implicit time increased, while the VEP amplitudes were not significantly different compared to those of controls [58].

## 10. Isotretinoin

Isotretinoin (13-cis-retinoic acid) is a vitamin A derivative that is commonly used in the treatment of severe refractory acne vulgaris by reducing sebum production [59]. As vitamin A plays a central role in the visual cycle, the use of isotretinoin has also been associated with impaired dark adaptation and night blindness [60]. Once a patient develops ocular symptoms, cessation of isotretinoin therapy is typically associated with recovery of night vision [61].

On ffERG, a patient who has visual toxicity associated with isotretinoin can be seen with reduced a-wave amplitude in scotopic conditions [60,62], reduced b-wave amplitude, and low a-wave to b-wave amplitude ratio [63]. The subclinical changes in ERG appear to persist even after cessation of the therapy and can be seen months or years after therapy [60,63]. EOG can be either normal or slightly subnormal with a reduced light-peak to dark-trough ratio in some patients with isotretinoin-associated visual toxicity [60,62]. In summary, electrophysiologic techniques, particularly ERG, may be used as a diagnostic tool in both diagnosing and monitoring isotretinoin-associated visual toxicity.

## 11. Ocular Siderosis

Ocular siderosis is a condition in which an iron-containing intraocular foreign body (IOFB) is retained in the eye, commonly following trauma. While large fragments are likely to cause intraocular hemorrhages or symptomatic cataracts that are managed surgically, retained smaller foreign bodies can result in ocular siderosis. Typical clinical signs include deposits beneath the anterior capsule, heterochromia, pupillary mydriasis, and iron deposits on the corneal endothelium [64]. Patients usually present 2–24 months after the inciting trauma and have reduced visual acuity.

On ffERG, patients with early stage ocular siderosis show reduced b-wave amplitude and lack of oscillatory potential [64]. However, if left untreated over years (i.e., lack of surgical removal of the IOFB), the reduction in ffERG amplitudes can further worsen and eventually become unrecordable [65]. After surgical treatment, ffERGs typically recover in response amplitudes, with the exception of oscillatory potentials [66]. Therefore, electrophysiologic testing can be a useful tool to not only diagnose but also help monitor the progression of patients with ocular siderosis.

## 12. Pentosan

Pentosan polysulfate sodium (PPS) is an oral medication currently approved for the treatment of interstitial cystitis. The association between PPS use and pigmentary maculopathy was first reported in 2018 in six patients [67]. While its exact mechanism remains uncertain, patients most commonly present with blurry vision, impaired subjective dark adaptation, and metamorphopsia [68]. PPS is characterized by hyperpigmented macular spots with pale yellow deposits bilaterally on fundus exam and RPE elevation and thickening on OCT. Fundus autofluorescence (FAF) also characteristically shows a pattern of hyperfluorescent and hypofluorescent spots in an array pattern that extends from the macula to the peripheral retina [68,69].

PPS-related maculopathy is also associated with changes in electrophysiology. On ffERG, cases have been reported to have normal ERG, borderline low cone-derived response amplitudes, or mildly attenuated rod-derived responses [67,68,69]. On mfERG, cases reported have ranged from normal to mild attenuation of response densities and delayed peak implicit times in central and pericentral areas, most prominently in rings 1–3 [67]. The EOG light-peak to dark-trough ratio was reported to be normal in three cases [69]. No studies to our knowledge have studied the VEP response to PPS-related maculopathy. Further studies on the electrophysiology of PPS-related maculopathy will help us further understand the mechanism of the toxicity.

## 13. PDE5 Inhibitors

Phosphodiesterases type 5 inhibitors (PDE5 inhibitors) are oral medications commonly used in the treatment of erectile dysfunction. Examples include medications such as sildenafil, tadalafil, and vardenafil. PDE5 inhibitors are theorized to cause visual side effects by weakly (10% relative effectiveness) inhibiting phosphodiesterase type 6, which is present on rods and cones and is a key component in the phototransduction cascade [70,71] Patients most commonly present with blue-tinged vision and increased sensitivity to light a few hours after receiving the medication [70].

PDE5-related visual changes can also be detected by electrophysiologic testing and may be related to the dose-dependent alternation of rod/cone functions by PDE-5 inhibitors [71]. On ffERG, reductions in photopic a-wave and b-wave amplitudes were seen 1 h after administration of sildenafil before returning to normal within 6 h [72]. The implicit times of photopic and 30 Hz flicker responses were also increased [71]. On mfERG, sildenafil is also associated with delayed and attenuated responses across the posterior pole [71]. Compared to the change in ERG, VEP appeared to be unchanged during clinical toxicity [72].

## 14. Phenothiazines (Chlorpromazine and Thioridazine)

Phenothiazines are a group of medicines for the treatment of schizophrenia and other psychotic disorders. Chlorpromazine and thioridazine are phenothiazine anti-psychotic medications that achieve their therapeutic effect by antagonism of postsynaptic dopamine receptors [73,74]. Among the phenothiazines, chlorpromazine and thioridazine have been studied extensively for their dose-dependent ocular side effects, which include pigmentation of eyelids, corneal edema, and pigmentary retinopathy [75]. Thioridazine has been associated with more cases of retinal toxicity, but there have also been a few cases reported for chlorpromazine [76,77]. Specifically, a dosage higher than 1000 mg/day of thioridazine for some weeks is associated with retinal toxicity [75]. The retinal toxicity may be related to the blockage of dopamine’s neuroprotective effect, leading to phototoxicity [75].

On ffERG, some studies have noted that chlorpromazine use is associated with reduced b-wave amplitudes [76,77], and delayed implicit times of ffERG, PERG, and pattern VEP [78]. However, in studies with schizophrenic patients who took chlorpromazine, no correlation between ERG a-wave amplitude and the dose of chlorpromazine was found [79]. On VEP, chlorpromazine is associated with increases in P100 implicit time [80].

Thioridazine, similarly, is associated with diminished b-wave amplitude, delayed a-wave implicit time, and delayed b-wave implicit time in scotopic ffERG [81]. On EOG, patients who received thioridazine showed either normal or reduced light-peak to dark-trough ratio [77] On VEP, similarly, thioridazine is associated with increased implicit times and decreased amplitudes [82].

## 15. Quinine

Quinine is a medication commonly used to treat malaria, with an additional off-label use for treating nocturnal leg cramps. It achieves its antimalarial effect by disruption of the nucleic acid and protein synthesis of the *P. falciparum* parasite, but it has a narrow therapeutic window with significant systemic toxicity in high plasma concentrations and common side effects including nausea, headache, and hypotension [83,84]. While the exact mechanism of quinine-related ocular toxicity remains unclear, the most common manifestations are loss of peripheral vision and a dramatic decrease in visual acuity early on in the acute toxicity phase despite the fundus appearing normal [85]. In a later stage, the retinal vessel may become attenuated, and optic disc pallor can develop.

Quinine toxicity is characterized by a reduction in ffERG a-wave and b-wave amplitudes in the acute phase. Later, the a-wave amplitude may partially or completely recover with persistent b-wave amplitude reduction, leading to an electronegative wave appearance [83,84,85,86]. On mfERG, the responses are attenuated in the whole tested field and are more severe peripherally compared to centrally [83,84]. On pattern reversal VEP, quinine toxicity is also associated with delayed implicit time [86,87]. EOG can appear to have reduced light-peak to dark-trough ratio in the acute toxicity phase before returning to normal [87,88].

## 16. Tamoxifen

Tamoxifen is a selective estrogen receptor modulator (SERM) that is commonly used in the treatment of estrogen receptor positive (ER+) breast cancer. Tamoxifen is associated with retinopathy characterized by refractile superficial crystalline deposits in the inner retina and punctate gray lesions in the retinal pigment epithelium and outer retina [89]. The parafoveal hyperreflective deposits can be also seen on OCT in an affected patient [90].

On multiple studies of both ffERG and mfERG, the patients treated with tamoxifen did not present significant changes compared to healthy controls in low- or high-dose tamoxifen, even with the refractile deposit visualized on fundus exam [91,92,93]. A few case reports reported that tamoxifen use is associated with slightly reduced a-wave and b-wave amplitude on ffERG [94]. On mfERG, there is a case report that tamoxifen caused a reduction in response densities in the aracentral and pericentral retina [95]. However, further investigation is needed to clarify the variable effect of tamoxifen on the electrophysiology of patients with different ocular diseases.

## 17. Topiramate

Topiramate is an anticonvulsant that is also used to treat migraines. It is a sulfa-derivative monosaccharide that achieves its effect by the enhancement of gamma-aminobutyric acid (GABA) transmission, blocking of neuronal voltage-gated sodium channels, and mild inhibition of carbonic anhydrase isoenzymes. It has well-known common ocular side effects, including inducing myopic shift and angle closure glaucoma [96,97]. In addition, it has some uncommon side effects, including retinal detachment and vitelliform maculopathy [98,99].

A patient with topiramate-related vitelliform maculopathy was reported to have an electronegative ffERG and normal EOG [99]. In rabbits treated with topiramate, ffERG showed reduced b-wave amplitudes to 30 Hz flicker [100]. While the exact mechanism remains uncertain, the electrophysiologic changes may be related to the modifications of ion transportation by topiramate on the voltage-gated sodium channels and GABAergic transmission that leads to the possible accumulation of GABA on the photoreceptor inner segment [99]. On VEP, topiramate treatment of migraine-without-aura patients appeared to not have a significant effect on the electrophysiologic response [101].

## 18. Vigabatrin

Vigabatrin (VGB) is an antiepileptic medication that achieves its effect by blocking the GABA degradation enzyme GABA-transaminase, irreversibly inhibiting the degradation of GABA [102,103]. It is an approved treatment for refractory complex partial seizures and infantile spasms. VGB is a structural analog of GABA and functions by irreversibly binding to the GABA transaminase, which increases the concentration of GABA in the central nervous system, including in the retina [104,105]. Studies also demonstrated that VGB has some impact in stimulating GABA release or inhibiting glial GABA uptake. The accumulation of GABA also results in cellular excitability that has been hypothesized to induce retinal excitotoxicity that eventually leads to retinal neuronal cell death [106]. The underlying mechanisms are associated with VGB-induced taurine deficiency [107] and the augmentation of the RRAG/mTOR pathway, [108] which can be enhanced by light exposure [109].

The first reported visual adverse effects from VGB were primarily peripheral visual field defects [110,111]. Patients can also present with reduced visual acuity and contrast sensitivity and have reduced nerve fiber layer thickness on optical coherence tomography (OCT) [112]. Patients who have been exposed to VGB also show electrophysiologic changes. On ffERG, the most common abnormalities seen include reduced b-wave amplitudes in both isolated rod response and mixed rod–cone response, 30 Hz flicker amplitude, and early oscillatory amplitudes [104,113,114]. These changes were seen in both adults and children who received VGB [115,116]. Specifically, the reduction in b-wave amplitude and the 30 Hz flicker ERG amplitude are considered to be the early signs of VGB toxicity. In addition, the 30 Hz flicker response in ffERG is most vulnerable to the VGB-induced retinal change and should be monitored regularly [111]. On mfERG, abnormal signals were found to correlate well with visual field deficits [117]. On EOG, a reduced light-peak to dark-trough ratio was also found, suggesting damage to the retinal pigment epithelium [106].

## 19. Vitamin A Deficiency

Vitamin A is a fat-soluble vitamin that is critical in the formation of rhodopsin from opsin, and its deficiency has a significant effect on vision. Vitamin A deficiency (VAD) can result in a variety of clinical presentations, including night blindness, xerophthalmia, growth and developmental disturbances, and an increased risk for severe infection [118]. VAD may be a result of poor nutrition, decreased intestinal absorption, or reduced storage due to liver pathologies [119]. Night blindness is one of the first signs of VAD, with other findings including conjunctival and corneal xerosis, Bitot’s spot, and xerophthalmic fundus that is characterized by white peripheral dots [120,121].

VAD has a characteristic pattern on ffERG. Patients with VAD have a complete absence of rod response bilaterally and reduced a-wave and b-wave amplitudes in cone responses to both 30 Hz flicker and single flash [119,120,122]. Abnormalities have also been noted in s-cone ERGs and PERGs [119]. On mfERG, macular responses are abnormal with diffusely diminished amplitudes [122].

With vitamin A supplementation, the visual deficits noted by patients with VAD can be reversed rather swiftly. The rod function and the generalized depression in cone function are returned to normal within 3 days, and s-cone amplitude and cone latencies can be recovered to normal within 12 days of supplementation [119].

## 20. Conclusions

This study summarizes some substances that can affect different visual electrophysiologic responses according to previous studies (Table 2). Different substances have different molecular mechanisms that may cause different combinations of abnormalities in ffERG, mfERG, PERG, VEP, and/or EOG. Combined with the other ocular exams, electrophysiologic exams which are more sensitive than other ocular exams in a few cases, provide additional objective functional indices for differential diagnosis, as well as for monitoring the side effects of some drugs on the retina and optic nerve.

With the development of new drugs and new studies of drugs’ adverse effects on the retina and optic nerve that can be reflected in changes in visual electrophysiologic responses, the content in this study can be expanded and updated in the future.

## Figures and Tables

**Table 1:** Generic vs. brand names for all study medications.

Generic	Brand
Amiodarone	Cordarone Nextarone Pacearone
Cefuroxime	Ceftin Kefurox Zinaceft
Cisplatin	Platinol
Desferoxamine, desferrioxamine	Desferal
Digoxin	Cardoxin Digitek Digox Lanoxicaps Lanoxin
Ethambutol	Myambutol
Hydroxychloroquine	Quineprox Plaquenil Plaquenil Sulfate
Isoretinoin	Absorica Accutane Amnesteem Claravis Myorisan Sotret Zenatane
Pentosan Polysulfate Sodium	Elmiron
Avanafil	Stendra
Sildenafil	Revatio Viagra
Tadalafil	Adcirca Cialis
Vardenafil	Levitra Staxyn
Chlorpromazine	Ormazine Thorazine Thorazine Spansule
Thioridazine	Mellaril
Quinine	QM-260 Qualaquin Quinamm
Tamoxifen	Nolvadex Soltamox
Topiramate	Eprontia Qudexy XR Topamax Trokendi XR Topiragen
Vigabatrin	Sabril Vigadrone

**Table 2:** Parameters of visual electrophysiologic responses that are affected by different substances *.

Substance	ffERG	mfERG	PERG	VEP	EOG	References
Alcohol	A−, I+			A−, I+		[3,6,8]
Amiodarone		A−				[12]
Cefuroxime	A−	A−				[13,14,17]
Cisplatin	A−			A−, I+	A−	[20,23,24,26]
Deferoxamine	A−, I+	A−, I+		I+	A−	[28,31,32,33]
Digoxin	A−, I+	A−	A−			[35,37,38]
Ethambutol	A−, I+			A−, I+		[43,44,45,50]
Hydroxychloroquine		A−, I+		I+		[53,54,56,57,58]
Isotretinoin	A−					[60,62]
Ocular siderosis	A−					[64,65]
Pentosane	A−	A−, I+				[67,68,69]
PDE5 Inhibitors	A−, I+	A−, I+				[71,72]
Phenothiazines	A−, I+		A−, I+	A−, I+	A−	[76,77,78,80,81,82]
Quinine	A−	A−		I+	A−	[83,84,85,86]
Tamoxifen		A−				[95]
Topiramate	A−					[99]
Vigabatrin	A−	A−			A−	[104,106,111,113,114,115,116,117]
Vitamin A deficiency	A−	A−	A−			[119,120,122]

* A: Amplitude or light-peak to dark-trough ratio; I: Implicit time. + Increase. − Decrease.

## Data Availability

Not applicable.

## References

[1] Karimi S., Arabi A., Shahraki T. (2021). Alcohol and the Eye. J. Ophthalmic. Vis. Res..

[2] Shin Y.W., Uhm K.B. (2011). A case of optic nerve atrophy with severe disc cupping after methanol poisoning. Korean J. Ophthalmol..

[3] Sharma R., Marasini S., Sharma A.K., Shrestha J.K., Nepal B.P. (2012). Methanol poisoning: Ocular and neurological manifestations. Optom. Vis. Sci..

[4] Xie X., Feng K., Wang J., Zhang M., Hong J., Zhang H. (2022). Comprehensive visual electrophysiological measurements discover crucial changes caused by alcohol addiction in humans: Clinical values in early prevention of alcoholic vision decline. Front. Neural Circuits.

[5] Liu D.M., Zhou S., Chen J.M., Peng S.Y., Xia W.T. (2016). The Intoxication Effects of Methanol and Formic Acid on Rat Retina Function. J. Ophthalmol..

[6] Urban P., Zakharov S., Diblik P., Pelclova D., Ridzon P. (2016). Visual evoked potentials in patients after methanol poisoning. Int. J. Occup. Med. Environ. Health.

[7] Stromland K. (1987). Ocular involvement in the fetal alcohol syndrome. Surv. Ophthalmol..

[8] Hug T.E., Fitzgerald K.M., Cibis G.W. (2000). Clinical and electroretinographic findings in fetal alcohol syndrome. J. Am. Assoc. Pediatr. Ophthalmol. Strabismus.

[9] Cohen-Lehman J., Dahl P., Danzi S., Klein I. (2010). Effects of amiodarone therapy on thyroid function. Nat. Rev. Endocrinol..

[10] Papiris S.A., Triantafillidou C., Kolilekas L., Markoulaki D., Manali E.D. (2010). Amiodarone: Review of pulmonary effects and toxicity. Drug Saf..

[11] Mantyjarvi M., Tuppurainen K., Ikaheimo K. (1998). Ocular side effects of amiodarone. Surv. Ophthalmol..

[12] Shaikh S., Shaikh N., Chun S.H., Spin J.M., Blumenkranz M.S., Marmor M.F. (2003). Retinal evaluation of patients on chronic amiodarone therapy. Retina.

[13] Ku J.Y., Wong S.W., Steeples L.R., Delaney C., Parry N.R.A., Fenerty C. (2022). High dose cefuroxime causing retinal toxicity in a patient undergoing trabeculectomy. Am. J. Ophthalmol. Case Rep..

[14] Delyfer M.N., Rougier M.B., Leoni S., Zhang Q., Dalbon F., Colin J., Korobelnik J.F. (2011). Ocular toxicity after intracameral injection of very high doses of cefuroxime during cataract surgery. J. Cataract Refract. Surg..

[15] Qureshi F., Clark D. (2011). Macular infarction after inadvertent intracameral cefuroxime. J. Cataract Refract. Surg..

[16] Diez-Alvarez L., Salva-Palomeque T., Jaumandreu L., Gomez-Mariscal M., Munoz-Negrete F.J., Rebolleda G. (2021). Ocular toxicity after inadvertent overdose of intracameral cefuroxime during cataract surgery. Arch. Soc. Esp. Oftalmol..

[17] Faure C., Perreira D., Audo I. (2015). Retinal toxicity after intracameral use of a standard dose of cefuroxime during cataract surgery. Doc. Ophthalmol..

[18] Wang M.Y., Arnold A.C., Vinters H.V., Glasgow B.J. (2000). Bilateral blindness and lumbosacral myelopathy associated with high-dose carmustine and cisplatin therapy. Am. J. Ophthalmol..

[19] Alkan A., Talaz S. (2019). Cilioretinal artery occlusion associated with cisplatin. J. Oncol. Pharm. Pract..

[20] Caraceni A., Martini C., Spatti G., Thomas A., Onofrj M. (1997). Recovering optic neuritis during systemic cisplatin and carboplatin chemotherapy. Acta Neurol. Scand..

[21] Kwan A.S., Sahu A., Palexes G. (2006). Retinal ischemia with neovascularization in cisplatin related retinal toxicity. Am. J. Ophthalmol..

[22] Yazici A., Sogutlu-Sari E., Yay A., Aksit H., Kilic A., Aksit D., Yildiz O., Ermis S.S. (2014). The protective effect of selenium in cisplatin-related retinotoxicity. Cutan. Ocul. Toxicol..

[23] Dulz S., Asselborn N.H., Dieckmann K.P., Matthies C., Wagner W., Weidmann J., Seidel C., Oing C., Berger L.A., Alsdorf W. (2017). Retinal toxicity after cisplatin-based chemotherapy in patients with germ cell cancer. J. Cancer Res. Clin. Oncol..

[24] Maiese K., Walker R.W., Gargan R., Victor J.D. (1992). Intra-arterial cisplatin--associated optic and otic toxicity. Arch. Neurol..

[25] Hilliard L.M., Berkow R.L., Watterson J., Ballard E.A., Balzer G.K., Moertel C.L. (1997). Retinal toxicity associated with cisplatin and etoposide in pediatric patients. Med. Pediatr. Oncol..

[26] Miller D.F., Bay J.W., Lederman R.J., Purvis J.D., Rogers L.R., Tomsak R.L. (1985). Ocular and orbital toxicity following intracarotid injection of BCNU (carmustine) and cisplatinum for malignant gliomas. Ophthalmology.

[27] Di Nicola M., Barteselli G., Dell’Arti L., Ratiglia R., Viola F. (2015). Functional and Structural Abnormalities in Deferoxamine Retinopathy: A Review of the Literature. BioMed Res. Int..

[28] Haimovici R., D’Amico D.J., Gragoudas E.S., Sokol S., Deferoxamine Retinopathy Study G. (2002). The expanded clinical spectrum of deferoxamine retinopathy. Ophthalmology.

[29] Lakhanpal V., Schocket S.S., Jiji R. (1984). Deferoxamine (Desferal)-induced toxic retinal pigmentary degeneration and presumed optic neuropathy. Ophthalmology.

[30] Davies S.C., Marcus R.E., Hungerford J.L., Miller M.H., Arden G.B., Huehns E.R. (1983). Ocular toxicity of high-dose intravenous desferrioxamine. Lancet.

[31] Dettoraki M., Kattamis A., Ladas I., Maragkos K., Koutsandrea C., Chatzistefanou K., Laios K., Brouzas D., Moschos M.M. (2017). Electrophysiological assessment for early detection of retinal dysfunction in beta-thalassemia major patients. Graefes Arch. Clin. Exp. Ophthalmol..

[32] Arden G.B., Wonke B., Kennedy C., Huehns E.R. (1984). Ocular changes in patients undergoing long-term desferrioxamine treatment. Br. J. Ophthalmol..

[33] Taylor M.J., Keenan N.K., Gallant T., Skarf B., Freedman M.H., Logan W.J. (1987). Subclinical VEP abnormalities in patients on chronic deferoxamine therapy: Longitudinal studies. Electroencephalogr. Clin. Neurophysiol..

[34] Economou M., Zafeiriou D.I., Kontopoulos E., Gompakis N., Koussi A., Perifanis V., Athanassiou-Metaxa M. (2006). Neurophysiologic and intellectual evaluation of beta-thalassemia patients. Brain Dev..

[35] Renard D., Rubli E., Voide N., Borruat F.X., Rothuizen L.E. (2015). Spectrum of digoxin-induced ocular toxicity: A case report and literature review. BMC Res. Notes.

[36] Lawrenson J.G., Kelly C., Lawrenson A.L., Birch J. (2002). Acquired colour vision deficiency in patients receiving digoxin maintenance therapy. Br. J. Ophthalmol..

[37] Piltz J.R., Wertenbaker C., Lance S.E., Slamovits T., Leeper H.F. (1993). Digoxin toxicity. Recognizing the varied visual presentations. J. Clin. Neuroophthalmol..

[38] Butler V.P., Odel J.G., Rath E., Wolin M.J., Behrens M.M., Martin T.J., Kardon R.H., Gouras P. (1995). Digitalis-induced visual disturbances with therapeutic serum digitalis concentrations. Ann. Intern. Med..

[39] Weleber R.G., Shults W.T. (1981). Digoxin retinal toxicity. Clinical and electrophysiological evaluation of a cone dysfunction syndrome. Arch. Ophthalmol..

[40] Lee E.J., Kim S.J., Choung H.K., Kim J.H., Yu Y.S. (2008). Incidence and clinical features of ethambutol-induced optic neuropathy in Korea. J. Neuroophthalmol..

[41] Chen S.C., Lin M.C., Sheu S.J. (2015). Incidence and prognostic factor of ethambutol-related optic neuropathy: 10-year experience in southern Taiwan. Kaohsiung J. Med. Sci..

[42] Vistamehr S., Walsh T.J., Adelman R.A. (2007). Ethambutol neuroretinopathy. Semin. Ophthalmol..

[43] Hennekes R. (1982). Clinical ERG findings in ethambutol intoxication. Graefes Arch. Clin. Exp. Ophthalmol..

[44] Williams D.E. (1984). Visual electrophysiology and psychophysics in chronic alcoholics and in patients on tuberculostatic chemotherapy. Am. J. Optom. Physiol. Opt..

[45] Kakisu Y., Adachi-Usami E., Mizota A. (1987). Pattern electroretinogram and visual evoked cortical potential in ethambutol optic neuropathy. Doc. Ophthalmol..

[46] Lai T.Y., Ngai J.W., Lai R.Y., Lam D.S. (2009). Multifocal electroretinography changes in patients on ethambutol therapy. Eye.

[47] Behbehani R.S., Affel E.L., Sergott R.C., Savino P.J. (2005). Multifocal ERG in ethambutol associated visual loss. Br. J. Ophthalmol..

[48] Lai T.Y., Chan W.M., Lam D.S., Lim E. (2005). Multifocal electroretinogram demonstrated macular toxicity associated with ethambutol related optic neuropathy. Br. J. Ophthalmol..

[49] Yen M.Y., Wang A.G., Chiang S.C., Liu J.H. (2000). Ethambutol retinal toxicity: An electrophysiologic study. J. Formos. Med. Assoc..

[50] Petrera J.E., Fledelius H.C., Trojaborg W. (1988). Serial pattern evoked potential recording in a case of toxic optic neuropathy due to ethambutol. Electroencephalogr. Clin. Neurophysiol..

[51] Rosenbaum J.T., Costenbader K.H., Desmarais J., Ginzler E.M., Fett N., Goodman S.M., O’Dell J.R., Schmajuk G., Werth V.P., Melles R.B. (2021). American College of Rheumatology, American Academy of Dermatology, Rheumatologic Dermatology Society, and American Academy of Ophthalmology 2020 Joint Statement on Hydroxychloroquine Use With Respect to Retinal Toxicity. Arthritis Rheumatol..

[52] Yusuf I.H., Sharma S., Luqmani R., Downes S.M. (2017). Hydroxychloroquine retinopathy. Eye.

[53] Tsang A.C., Ahmadi Pirshahid S., Virgili G., Gottlieb C.C., Hamilton J., Coupland S.G. (2015). Hydroxychloroquine and chloroquine retinopathy: A systematic review evaluating the multifocal electroretinogram as a screening test. Ophthalmology.

[54] Maturi R.K., Yu M., Weleber R.G. (2004). Multifocal electroretinographic evaluation of long-term hydroxychloroquine users. Arch. Ophthalmol..

[55] Adamptey B., Rudnisky C.J., MacDonald I.M. (2020). Effect of stopping hydroxychloroquine therapy on the multifocal electroretinogram in patients with rheumatic disorders. Can. J. Ophthalmol..

[56] Chang W.H., Katz B.J., Warner J.E., Vitale A.T., Creel D., Digre K.B. (2008). A novel method for screening the multifocal electroretonogram in patients using hydroxychloroquine. Retina.

[57] Nebbioso M., Livani M.L., Steigerwalt R.D., Panetta V., Rispoli E. (2011). Retina in rheumatic diseases: Standard full field and multifocal electroretinography in hydroxychloroquine retinal dysfunction. Clin. Exp. Optom..

[58] Heravian J., Saghafi M., Shoeibi N., Hassanzadeh S., Shakeri M.T., Sharepoor M. (2011). A comparative study of the usefulness of color vision, photostress recovery time, and visual evoked potential tests in early detection of ocular toxicity from hydroxychloroquine. Int. Ophthalmol..

[59] Leyden J.J., Del Rosso J.Q., Baum E.W. (2014). The use of isotretinoin in the treatment of acne vulgaris: Clinical considerations and future directions. J. Clin. Aesthet. Dermatol..

[60] Weleber R.G., Denman S.T., Hanifin J.M., Cunningham W.J. (1986). Abnormal retinal function associated with isotretinoin therapy for acne. Arch. Ophthalmol..

[61] Teo K., Yazdabadi A. (2014). Isotretinoin and night blindness. Australas J. Dermatol.

[62] Brown R.D., Grattan C.E. (1989). Visual toxicity of synthetic retinoids. Br. J. Ophthalmol.

[63] Mollan S.P., Woodcock M., Siddiqi R., Huntbach J., Good P., Scott R.A. (2006). Does use of isotretinoin rule out a career in flying?. Br. J. Ophthalmol.

[64] Hope-Ross M., Mahon G.J., Johnston P.B. (1993). Ocular siderosis. Eye.

[65] Schechner R., Miller B., Merksamer E., Perlman I. (1990). A long term follow up of ocular siderosis: Quantitative assessment of the electroretinogram. Doc. Ophthalmol..

[66] Imaizumi M., Matsumoto C.S., Yamada K., Nanba Y., Takaki Y., Nakatsuka K. (2000). Electroretinographic assessment of early changes in ocular siderosis. Ophthalmologica.

[67] Pearce W.A., Chen R., Jain N. (2018). Pigmentary Maculopathy Associated with Chronic Exposure to Pentosan Polysulfate Sodium. Ophthalmology.

[68] Hanif A.M., Armenti S.T., Taylor S.C., Shah R.A., Igelman A.D., Jayasundera K.T., Pennesi M.E., Khurana R.N., Foote J.E., O’Keefe G.A. (2019). Phenotypic Spectrum of Pentosan Polysulfate Sodium-Associated Maculopathy: A Multicenter Study. JAMA Ophthalmol..

[69] Abou-Jaoude M.M., Davis A.M., Fraser C.E., Leys M., Hinkle D., Odom J.V., Maldonado R.S. (2021). New Insights Into Pentosan Polysulfate Maculopathy. Ophthalmic Surg. Lasers Imaging Retin..

[70] Kerr N.M., Danesh-Meyer H.V. (2009). Phosphodiesterase inhibitors and the eye. Clin. Exp. Ophthalmol..

[71] Luu J.K., Chappelow A.V., McCulley T.J., Marmor M.F. (2001). Acute effects of sildenafil on the electroretinogram and multifocal electroretinogram. Am. J. Ophthalmol..

[72] Vobig M.A., Klotz T., Staak M., Bartz-Schmidt K.U., Engelmann U., Walter P. (1999). Retinal side-effects of sildenafil. Lancet.

[73] Tamminga C.A., Carlsson A. (2002). Partial dopamine agonists and dopaminergic stabilizers, in the treatment of psychosis. Curr. Drug Targets CNS Neurol. Disord..

[74] Meltzer H.Y., Sachar E.J., Frantz A.G. (1975). Dopamine antagonism by thioridazine in schizophrenia. Biol. Psychiatry.

[75] Richa S., Yazbek J.C. (2010). Ocular adverse effects of common psychotropic agents: A review. CNS Drugs.

[76] Holopigian K., Clewner L., Seiple W., Kupersmith M.J. (1994). The effects of dopamine blockade on the human flash electroretinogram. Doc. Ophthalmol..

[77] Meredith T.A., Aaberg T.M., Willerson W.D. (1978). Progressive chorioretinopathy after receiving thioridazine. Arch. Ophthalmol..

[78] Bartel P., Blom M., Robinson E., Van der Meyden C., Sommers D.O., Becker P. (1990). Effects of chlorpromazine on pattern and flash ERGs and VEPs compared to oxazepam and to placebo in normal subjects. Electroencephalogr. Clin. Neurophysiol..

[79] Warner R., Laugharne J., Peet M., Brown L., Rogers N. (1999). Retinal function as a marker for cell membrane omega-3 fatty acid depletion in schizophrenia: A pilot study. Biol. Psychiatry.

[80] Bartel P., Blom M., van der Meyden C., de Klerk S. (1988). Effects of single doses of diazepam, chlorpromazine, imipramine and trihexyphenidyl on visual-evoked potentials. Neuropsychobiology.

[81] Filip V., Balik J. (1978). Possible indication of dopaminergic blockade in man by electroretinography. Int. Pharm..

[82] Saletu B., Saletu M., Simeon J., Viamontes G., Itil T.M. (1975). Comparative symptomatological and evoked potential studies with d-amphetamine, thioridazine, and placebo in hyperkinetic children. Biol. Psychiatry.

[83] Audo I., Robson A.G., Holder G.E., Moore A.T. (2008). The negative ERG: Clinical phenotypes and disease mechanisms of inner retinal dysfunction. Surv. Ophthalmol..

[84] Verdon W. (2008). Clinical electrophysiology in quinine induced retinal toxicity. Optom. Vis. Sci..

[85] Freund P.R., Wright T., Margolin E.A. (2020). Toxic Optic Neuropathy From Quinine Overdose. J. Neuroophthalmol..

[86] Saeed M.U., Noonan C., Hagan R., Brown M. (2011). Relatively spared central multifocal electroretinogram responses in acute quinine toxicity. BMJ Case Rep..

[87] Gangitano J.L., Keltner J.L. (1980). Abnormalities of the pupil and visual-evoked potential in quinine amblyopia. Am. J. Ophthalmol..

[88] Canning C.R., Hague S. (1988). Ocular quinine toxicity. Br. J. Ophthalmol..

[89] Arora S., Surakiatchanukul T., Arora T., Errera M.H., Agrawal H., Lupidi M., Chhablani J. (2022). Retinal toxicities of systemic anticancer drugs. Surv. Ophthalmol..

[90] Caramoy A., Scholz P., Fauser S., Kirchhof B. (2011). Imaging tamoxifen retinopathy using spectral-domain optical coherence tomography. GMS Ophthalmol. Cases.

[91] Salomao S.R., Watanabe S.E., Berezovsky A., Motono M. (2007). Multifocal electroretinography, color discrimination and ocular toxicity in tamoxifen use. Curr. Eye Res..

[92] Watanabe S.E., Berezovsky A., Motono M., Sacai P.Y., Pereira J.M., Sallum J.M., Gebrim L.H., Salomao S.R. (2010). Retinal function in patients treated with tamoxifen. Doc. Ophthalmol..

[93] Bentley C.R., Davies G., Aclimandos W.A. (1992). Tamoxifen retinopathy: A rare but serious complication. BMJ.

[94] McKeown C.A., Swartz M., Blom J., Maggiano J.M. (1981). Tamoxifen retinopathy. Br. J. Ophthalmol..

[95] Hu Y., Liu N., Chen Y. (2017). The optical imaging and clinical features of tamoxifen associated macular hole: A case report and review of the literatures. Photodiagn. Photodyn. Ther..

[96] Hesami O., Hosseini S.S., Kazemi N., Hosseini-Zijoud S.M., Moghaddam N.B., Assarzadegan F., Mokhtari S., Fakhraee S. (2016). Evaluation of Ocular Side Effects in the Patients on Topiramate Therapy for Control of Migrainous Headache. J. Clin. Diagn. Res..

[97] Abtahi M.A., Abtahi S.H., Fazel F., Roomizadeh P., Etemadifar M., Jenab K., Akbari M. (2012). Topiramate and the vision: A systematic review. Clin. Ophthalmol..

[98] Rosenberg K., Maguire J., Benevento J. (2017). Topiramate-induced macular neurosensory retinal detachment. Am. J. Ophthalmol. Case Rep..

[99] Tsui I., Casper D., Chou C.L., Tsang S.H. (2008). Electronegative electroretinogram associated with topiramate toxicity and vitelliform maculopathy. Doc. Ophthalmol..

[100] Kjellstrom S., Bruun A., Isaksson B., Eriksson T., Andreasson S., Ponjavic V. (2006). Retinal function and histopathology in rabbits treated with Topiramate. Doc. Ophthalmol..

[101] De Luca C., Gori S., Mazzucchi S., Dini E., Cafalli M., Siciliano G., Papa M., Baldacci F. (2021). Supersaturation of VEP in Migraine without Aura Patients Treated with Topiramate: An Anatomo-Functional Biomarker of the Disease. J. Clin. Med..

[102] Yee J.M., Agulian S., Kocsis J.D. (1998). Vigabatrin enhances promoted release of GABA in neonatal rat optic nerve. Epilepsy Res..

[103] Maciel C.B., Teixeira F.J.P., Dickinson K.J., Spana J.C., Merck L.H., Rabinstein A.A., Sergott R., Shan G., Miao G., Peloquin C.A. (2022). Early vigabatrin augmenting GABA-ergic pathways in post-anoxic status epilepticus (VIGAB-STAT) phase IIa clinical trial study protocol. Neurol. Res. Pract..

[104] Foroozan R. (2018). Vigabatrin: Lessons Learned From the United States Experience. J. Neuroophthalmol..

[105] Ben-Menachem E. (2011). Mechanism of action of vigabatrin: Correcting misperceptions. Acta Neurol. Scand. Suppl..

[106] Heim M.K., Gidal B.E. (2012). Vigabatrin-associated retinal damage: Potential biochemical mechanisms. Acta Neurol. Scand..

[107] Tao Y., Yang J., Ma Z., Yan Z., Liu C., Ma J., Wang Y., Yang Z., Huang Y.F. (2016). The Vigabatrin Induced Retinal Toxicity is Associated with Photopic Exposure and Taurine Deficiency: An In Vivo Study. Cell Physiol. Biochem..

[108] Vogel K.R., Ainslie G.R., Schmidt M.A., Wisor J.P., Gibson K.M. (2017). mTOR Inhibition Mitigates Molecular and Biochemical Alterations of Vigabatrin-Induced Visual Field Toxicity in Mice. Pediatr. Neurol..

[109] Yang J., Naumann M.C., Tsai Y.T., Tosi J., Erol D., Lin C.S., Davis R.J., Tsang S.H. (2012). Vigabatrin-induced retinal toxicity is partially mediated by signaling in rod and cone photoreceptors. PLoS ONE.

[110] Daneshvar H., Racette L., Coupland S.G., Kertes P.J., Guberman A., Zackon D. (1999). Symptomatic and asymptomatic visual loss in patients taking vigabatrin. Ophthalmology.

[111] Sergott R.C., Westall C.A. (2011). Primer on visual field testing, electroretinography, and other visual assessments for patients treated with vigabatrin. Acta Neurol. Scand. Suppl..

[112] Clayton L.M., Devile M., Punte T., de Haan G.J., Sander J.W., Acheson J.F., Sisodiya S.M. (2012). Patterns of peripapillary retinal nerve fiber layer thinning in vigabatrin-exposed individuals. Ophthalmology.

[113] Morong S., Westall C.A., Nobile R., Buncic J.R., Logan W.J., Panton C.M., Abdolell M. (2003). Longitudinal changes in photopic OPs occurring with vigabatrin treatment. Doc. Ophthalmol..

[114] McDonagh J., Stephen L.J., Dolan F.M., Parks S., Dutton G.N., Kelly K., Keating D., Sills G.J., Brodie M.J. (2003). Peripheral retinal dysfunction in patients taking vigabatrin. Neurology.

[115] Kjellstrom U., Andreasson S., Ponjavic V. (2011). Electrophysiological evaluation of retinal function in children receiving vigabatrin medication. J. Pediatr. Ophthalmol. Strabismus.

[116] Moskowitz A., Hansen R.M., Eklund S.E., Fulton A.B. (2012). Electroretinographic (ERG) responses in pediatric patients using vigabatrin. Doc. Ophthalmol..

[117] Harding G.F., Wild J.M., Robertson K.A., Lawden M.C., Betts T.A., Barber C., Barnes P.M. (2000). Electro-oculography, electroretinography, visual evoked potentials, and multifocal electroretinography in patients with vigabatrin-attributed visual field constriction. Epilepsia.

[118] Sommer A. (2008). Vitamin a deficiency and clinical disease: An historical overview. J. Nutr..

[119] McBain V.A., Egan C.A., Pieris S.J., Supramaniam G., Webster A.R., Bird A.C., Holder G.E. (2007). Functional observations in vitamin A deficiency: Diagnosis and time course of recovery. Eye.

[120] Genead M.A., Fishman G.A., Lindeman M. (2009). Fundus white spots and acquired night blindness due to vitamin A deficiency. Doc. Ophthalmol..

[121] Gilbert C. (2013). The eye signs of vitamin A deficiency. Community Eye Health.

[122] Singer J.R., Bakall B., Gordon G.M., Reddy R.K. (2016). Treatment of vitamin A deficiency retinopathy with sublingual vitamin A palmitate. Doc. Ophthalmol..

